# Area of ischemia assessed by physicians and software packages from myocardial perfusion scintigrams

**DOI:** 10.1186/1471-2342-14-5

**Published:** 2014-01-31

**Authors:** Lars Edenbrandt, Peter Höglund, Sophia Frantz, Philip Hasbak, Allan Johansen, Lena Johansson, Annett Kammeier, Oliver Lindner, Milan Lomsky, Shinro Matsuo, Kenichi Nakajima, Karin Nyström, Eva Olsson, Karl Sjöstrand, Sven-Eric Svensson, Hiroshi Wakabayashi, Elin Trägårdh

**Affiliations:** 1 Clinical Physiology and Nuclear Medicine, Skåne University Hospital, Lund University, Inga Marie Nilssons gata 49, 205 02 Malmö, Sweden; 2 Department of Molecular and Clinical Medicine, Clinical Physiology, Sahlgrenska University Hospital, Gothenburg, Sweden; 3 EXINI Diagnostics AB, Lund, Sweden; 4 Competence Center for Clinical Research, Skåne University Hospital, Lund, Sweden; 5 Department of Clinical Physiology and Nuclear Medicine, Copenhagen University Hospital, Rigshospitalet, Copenhagen, Denmark; 6 Department of Nuclear Medicine, Odense University Hospital, Odense, Denmark; 7 Institute of Radiology, Nuclear Medicine and Molecular Imaging, Heart and Diabetes Center North Rhine-Westphalia, University Hospital of Ruhr University Bochum, Bad Oeynhausen, Germany; 8 Department of Nuclear Medicine, Kanazawa University, Kanazawa, Japan; 9 Department of Medical and Health Sciences, Linköping University and Department of Clinical Physiology, County Council of Östergötland, Linköping, Sweden; 10 Department of Clinical Physiology, Blekingesjukhuset, Karlskrona, Sweden

**Keywords:** Ischemic heart disease, Myocardial perfusion imaging, Intra-observer variability, Software tools

## Abstract

**Background:**

The European Society of Cardiology recommends that patients with >10% area of ischemia should receive revascularization. We investigated inter-observer variability for the extent of ischemic defects reported by different physicians and by different software tools, and if inter-observer variability was reduced when the physicians were provided with a computerized suggestion of the defects.

**Methods:**

Twenty-five myocardial perfusion single photon emission computed tomography (SPECT) patients who were regarded as ischemic according to the final report were included. Eleven physicians in nuclear medicine delineated the extent of the ischemic defects. After at least two weeks, they delineated the defects again, and were this time provided a suggestion of the defect delineation by EXINI Heart^TM^ (EXINI). Summed difference scores and ischemic extent values were obtained from four software programs.

**Results:**

The median extent values obtained from the 11 physicians varied between 8% and 34%, and between 9% and 16% for the software programs. For all 25 patients, mean extent obtained from EXINI was 17.0% (± standard deviation (SD) 14.6%). Mean extent for physicians was 22.6% (± 15.6%) for the first delineation and 19.1% (± 14.9%) for the evaluation where they were provided computerized suggestion. Intra-class correlation (ICC) increased from 0.56 (95% confidence interval (CI) 0.41-0.72) to 0.81 (95% CI 0.71-0.90) between the first and the second delineation, and SD between physicians were 7.8 (first) and 5.9 (second delineation).

**Conclusions:**

There was large variability in the estimated ischemic defect size obtained both from different physicians and from different software packages. When the physicians were provided with a suggested delineation, the inter-observer variability decreased significantly.

## Background

Myocardial perfusion scintigraphy (MPS) is widely regarded as a clinically useful non-invasive imaging method for the diagnosis of suspected coronary artery disease, identification of culprit lesions and risk assessment [1-4]. Visual interpretation of MPS studies is dependent on the knowledge of the physician, and subject to inter- and intra-observer variability. Software packages for automated quantification of MPS, such as ejection fraction and summed stress score (SSS), have been developed in order to make the interpretations more standardized.

Recent studies have shown that patients with significant ischemia and without extensive scar were likely to realize a survival benefit from early revascularization [3,5]. In contrast, the survival of patients with minimal ischemia was superior with medical therapy without early revascularization. Therefore, determination of the amount of ischemia is essential for patient management. In the studies, the percent ischemic myocardium was calculated using 5 point/20-segment MPS scoring, i.e. taking into account both the extent and severity of the ischemic myocardium. The results from the studies were recently incorporated into guidelines on revascularization. The European Society of Cardiology recommends that patients with stable angina or silent ischemia with “proven large area of ischemia (>10%)” should receive revascularization (class 1B) [6].

Several studies have demonstrated that the variability between different software packages to calculate scoring values are considerable [7-9], which will affect the calculated percent ischemic myocardium.

It has previously been shown that referring physicians to MPS tend to underestimate the extent of ischemia and infarction reported by the physician in nuclear medicine [10]. Because of the new recommendations, it is important to report the amount of the ischemic myocardium, preferably as a percentage of the left ventricle. It is also important that there is a consensus among physicians interpreting MPS images regarding the amount of the ischemic myocardium.

In this study we wanted to investigate 1) the inter-observer variability for the extent of reversible perfusion defects reported by different physicians in nuclear medicine, 2) the variability for the reversible perfusion defects obtained from different software tools, 3) the differences between the assessments made by the physicians and the different software packages and 4) if the inter-observer variability is reduced when the physicians are provided with a computerized suggestion of the delineation of the reversible perfusion defects.

## Methods

### Patients and MPS protocol

The study was approved by the local research ethics committee at Lund University and complies with the Declaration of Helsinki.

Twenty-five patients who underwent MPS in Malmö, Sweden January-February 2006 were included. The patients were chosen in a consecutive manner based on the final report, and only patients who where regarded as ischemic were included. 16 of the patients were male; 9 were stressed using maximal exercise test and 16 using Adenosine; mean age was 71 ± 8.5 years; mean ejection fraction 57 ± 11%; mean end-diastolic volume 139 ± 48 ml; 9 patients got the diagnosis acute coronary syndrome after the MPS (follow-up until 2010). According to the final report, 22 patients had single-vessel disease, 2 had multi-vessel disease and 1 had suspected multi-vessel disease.

The MPS studies were performed according to clinical routine, using a 2-day gated stress/gated rest ^99m^Tc-tetrofosmin protocol, starting with an injection of 600 MBq ^99m^Tc-tetrofosmin at stress. Patients were stressed using either maximal exercise on an ergometer or a pharmacological test with adenosine. The exercise test was continued for at least 1 min after the injection of the tracer and the adenosine infusion for at least 2 min after the injection of the tracer. This examination was followed by a rest study with injection of 600 MBq ^99m^Tc-tetrofosmin.

Stress and rest acquisition began 60 min after the end of the injection of ^99m^Tc-tetrofosmin. Images were obtained according to established clinical protocols, using single photon emission computed tomography over 180° elliptical, autocontour rotations from the 45° right anterior oblique position, with a dual-head gamma camera, e.cam (Siemens AG Medical Solutions, Erlangen, Germany). Patients were imaged in the supine position. A low energy high-resolution collimator and a zoom factor of 1.0 were used. We obtained 64 (32 views per camera) projections in a 128 × 128 matrix, with an acquisition time of 20 s per projection. Stress images were gated to the electrocardiogram using 8 frames per cardiac cycle. No automatic motion-correction program was applied; instead the acquisition was repeated if motion was detected. Tomographic reconstruction and calculation of short and long axis slice images were performed using e.soft (Siemens AG Medical Solutions, Erlangen, Germany). Images were reconstructed with filtered back-projection. A 2D Butterworth pre-reconstruction filter was used with cut-off frequency of 0.45 Nyquist frequency, order 5.

### Evaluations made by physicians

Eleven physicians in nuclear medicine from 8 different hospitals in 4 countries (Sweden, Denmark, Germany, and Japan) were invited to participate. Two of the physicians had 5 and 7 years of experience with interpreting MPS, and the other physicians had 15-40 years of experience. All cases were classified visually, and a custom display software was developed for this purpose, allowing the physicians to view non-attenuation corrected slice images (short axis, horizontal and vertical long axis) of the rest and stress studies, polar plots (rest, stress, rest-stress differences, stress/rest ratio) and 3D-images. Colour scales and contrast levels were adjustable. No quantitative results from the software package were available during the evaluation. The physicians were instructed to delineate the ischemic area in the polar plot. They could add as many ischemic areas as they wanted. The physicians were informed that the 25 cases had the diagnosis “reversible ischemia” according to the clinical report (in which all available clinical data, stress test, MPS study, etc. were considered) and the gender of the patient. No other information was given to the physicians (i.e. no information about known cardiac diseases, symptoms, electrocardiogram, stress test results, weight, etc.).

After at least two weeks, all physicians delineated the reversible defects again. This time they were provided a suggestion of the defect delineation by EXINI Heart^TM^ (EXINI Diagnostics AB, Sweden; EXINI), but otherwise the layout of the program was the same as before. The physicians were able to adjust the delineation suggested by EXINI, or they could remove the suggested delineation and create a new delineation. The studies were presented in a different randomized order, compared with the order in the first evaluation. Originally, none of the software packages had the feature for the physician to easily adjust a proposed delineation of a perfusion defect. We therefore developed that on the platform of EXINI and choose to use EXINI as reference method in the paper.

### Software packages

Four different software packages were compared; (EXINI version 5.0beta [11], Emory Cardiac Toolbox version 3.0 (Emory University; ECT [12]), Quantitative Perfusion SPECT version 4.0 (Cedars Sinai; QPS [13]), 4D-MSPECT version 4.0 (Invia Medical Solutions; 4DM [14]). Summed difference scores (SDS) were obtained from all 4 programs and compared to the total possible score from 17 segments (% ischemic myocardium); called EXINI summed difference % (SD%), ECT (SD%), QPS (SD%) and 4DM (SD%) in this article. This approach was included based on the study by Hachamovitch et al [3] and this measurement takes into account both the extent and the severity of the defects. The extent of the ischemic defects were also obtained from the programs; called EXINI (extent), ECT (extent), QPS (extent) and 4DM (extent) in this article. These measurements also reflect the percentage ischemic myocardium (extent/area), but are calculated using other methods compared to the SD% method. This approach was included due to the recommendations in revascularization guidelines [6].

### Automatic delineation of ischemic regions by EXINI

The automatic delineation of defects in EXINI proceeds in two stages. In stage one, defects in the stress image are delineated. From a number of seed points extracted from local intensity minima, deformable models of circular topology based on the active contour framework [15] are fit to the image based on edge and intensity information. This produces a set of “stress defects” which may be either reversible and/or irreversible. In a second phase, each stress defect is analysed in order to find strictly reversible sub-regions based on information from the difference image. Regions which are contained inside the stress defects and which exhibit high difference intensities are delineated using a simple smoothing and thresholding approach, producing a set of reversible “difference defects”. This paper concerns difference defects only.

### Statistical analysis

Descriptive statistics for continuous variables are given as mean and standard deviation (SD) or median and range. Inter-observer variability is expressed as intra-class correlation (ICC) with 95% confidence intervals (CI) between observers as estimated from linear mixed model analysis where patients and observers were entered as random effects. For comparison between the different programs and observers, linear mixed models were used where program (one at a time) and observers were entered as fixed effects and patients as random effects. For a comparison within observers’ mean values between the two occasions without and with EXINI, Wilcoxon’s signed rank test was used. The level of statistical significance was set at p < 0.05. Bland-Altman plots were created for analysis of relationship between the evaluations made by EXINI and the physicians, where EXINI was used as reference method [16].

## Results

### Inter-observer variability for physicians

The median values obtained for each of the 11 physicians for the extent of the ischemic defects varied between 8% and 34%. For each of the 25 studies, there was a large variation in the values reported by the different physicians. The median values and range reported by the physicians for each patient are shown in the last column of Table 1.

**Table 1:** The amount ischemic myocardium (%), extent and SD%, obtained by the different programs for the 25 patients

**Patient #**	**EXINI**	**EXINI**	**ECT**	**ECT**	**QPS**	**QPS**	**4DM**	**4DM**	**Physicians**
	**(extent)**	**(SD%)**	**(extent)**	**(SD%)**	**(extent)**	**(SD%)**	**(extent)**	**(SD%)**	
**1**	27	16	3	6	15	3	14	6	26 (8-65)
**2**	0	6	0	0	0	0	0	0	6 (1-25)
**3**	9	7	3	7	7	4	2	3	14 (6-22)
**4**	19	13	18	7	24	19	15	7	23 (15-34)
**5**	8	10	14	15	2	3	7	3	25 (3-34)
**6**	21	24	37	26	37	35	61	40	27 (12-41)
**7**	4	15	1	1	5	4	5	15	10 (4-29)
**8**	0	6	17	7	6	10	24	12	6 (0-22)
**9**	17	16	16	10	18	12	25	12	24 (14-31)
**10**	17	10	10	1	20	13	3	3	17 (4-31)
**11**	26	18	7	6	9	9	19	13	25 (2-43)
**12**	3	7	4	6	8	7	13	9	7 (2-17)
**13**	22	22	15	6	15	15	29	24	24 (15-41)
**14**	59	34	12	15	0	0	14	13	54 (27-74)
**15**	9	10	32	16	9	9	22	16	16 (2-32)
**16**	0	9	3	6	5	6	12	12	12 (0-26)
**17**	16	13	11	7	4	3	10	6	30 (10-42)
**18**	14	10	10	3	5	9	11	10	28 (11-43)
**19**	43	32	26	22	32	19	35	18	43 (24-66)
**20**	11	9	6	10	10	12	9	6	14 (3-22)
**21**	38	24	34	21	21	19	34	22	46 (17-61)
**22**	34	26	15	0	9	10	17	16	38 (14-73)
**23**	7	4	3	6	14	6	9	3	7 (3-24)
**24**	5	7	5	4	4	3	8	3	9 (1-29)
**25**	16	18	12	9	13	12	17	13	22 (9-36)
**Mean**	17.0	14.7	12.5	8.8	11.7	9.7	16.6	11.4	
**±SD**	14.6	8.2	10.4	6.9	9.4	7.7	13.1	8.6	

Median extent values (and range) obtained from the 11 physicians for each patient (without suggestion of delineation from EXINI) are given as a reference.

EXINI (extent): extent (%) of ischemic defect.

EXINI (SD%): amount (%) of ischemic defect based on 5-point/17-segment model.

### Variability between programs

The median values for the amount of ischemic area detected by the different programs were 16% (range 0-59%) for EXINI (extent), 13% (4-34%) for EXINI (SD%), 11% (0-37%) for ECT (extent), 7% (0-26%) for ECT (SD%), 9% (0-37%) for QPS (extent), 9% (0-35%) for QPS (SD%), 14% (0-61%) for 4DM (extent) and 12% (0-40%) for 4DM (SD%). There was a considerable variation in the reported amount of ischemic myocardium between the different programs for the 25 patients (Table 1). Table 2 shows the differences in mean ischemic area for the different software packages compared to EXINI (extent). The differences between EXINI (extent) and 4DM (SD%), QPS (extent), QPS (SD%), and ECT (SD%) were found to be significant (Table 2).

**Table 2:** Differences in mean ischemic area between EXINI and the other software packages

**EXINI (extent) vs:**	**Differences in mean ischemic area**	**P-value**	**Mean ischemic area ± SD (%)**
**EXINI (SD%)**	-2.4	0.75	14.7 (± 8.2)
**4DM (extent)**	-0.4	0.99	16.6 (± 13.1)
**4DM (SD%)**	-5.6	0.023	11.4 (± 8.6)
**ECT (extent)**	-4.4	0.12	12.5 (± 10.4)
**ECT (SD%)**	-8.3	<0.001	8.8 (± 6.7)
**QPS (extent)**	-5.3	0.036	11.7 (± 9.4)
**QPS (SD%)**	-7.3	<0.001	9.7 (± 7.7)

The right columns shows mean ischemic area ± SD (%) of the respective software packages.

EXINI (extent): extent (%) of ischemic defect.

EXINI (SD%): amount (%) of ischemic defect based on 5-point/17-segment model.

### Comparison between software packages and physicians

Table 3 shows the differences in mean ischemic extent values between the different physicians and the different programs. The only physician that had <5 percentage points differences between his/her evaluation and all of the software packages was physician #H. Two physicians (# C and D) had 5-10 percentage points larger ischemic areas compared to all programs. These physicians were located at the same hospital. Four physicians (# E, F, I and J) had at least 10 percentage points larger ischemic areas compared to all programs. These physicians were located at three different hospitals in two countries. For each program, the differences between the program and at least one of the physicians were <2 percentage points. Physicians # B and K had least experience (7 and 5 years) in interpreting MPS.

**Table 3:** Differences in mean extent values between each software package and each physician

**Physician**	**EXINI (extent)**	**EXINI (SD%)**	**4DM (extent)**	**4DM (SD%)**	**ECT (extent)**	**ECT (SD%)**	**QPS (extent)**	**QPS (SD%)**
**A**	4.2	6.5	4.6	9.8	8.6	12.5	9.5	11.5
**B**	-0.1	2.2	0.3	5.5	4.3	8.2	5.2	7.2
**C**	7.4	9.8	7.8	13.0	11.9	15.8	12.8	14.8
**D**	7.2	9.6	7.6	12.8	11.7	15.6	12.6	14.6
**E**	11.2	13.6	11.7	16.9	15.7	19.6	16.6	18.6
**F**	12.6	14.9	13.0	18.2	17.0	20.9	17.9	19.9
**G**	-2.1	0.2	-1.8	3.4	2.3	6.2	3.2	5.2
**H**	-3.4	-1.1	-3.1	2.1	1.0	4.8	1.8	3.8
**I**	12.6	14.9	13.0	18.2	17.0	20.9	17.9	19.9
**J**	19.1	21.4	19.5	24.7	23.6	27.4	24.4	26.4
**K**	-7.0	-4.6	-6.6	-1.4	-2.6	1.3	-1.7	0.3

EXINI (extent): extent (%) of ischemic defect.

EXINI (SD%): amount (%) of ischemic defect based on 5-point/17-segment model.

### Comparison of extent values obtained by physicians without and with computerized suggestion

**Figure 1:**
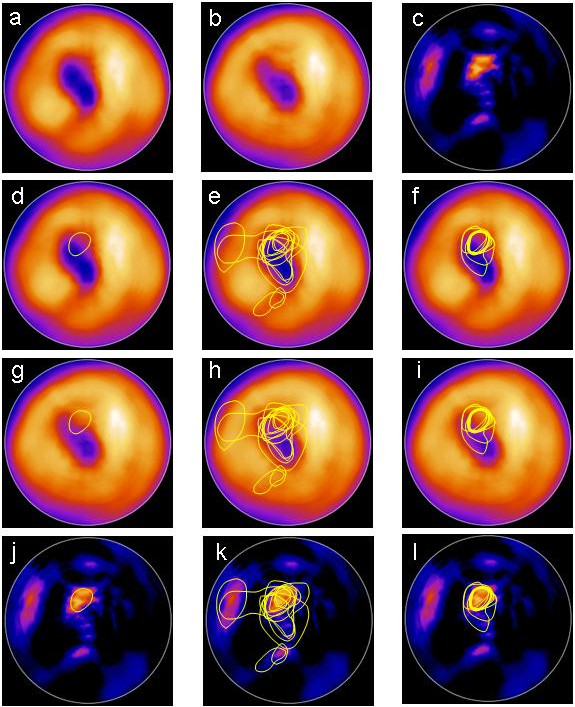
**The delineations of the ischemic area made by the physicians and by EXINI for one of the patients.** The upper row shows the stress **(a)** and rest **(b)** polar plots as well as the difference rest-stress plot **(c)**. The second row shows the delineation made by EXINI **(d)**, the delineations made by the 11 physicians without **(e)** and with **(f)** suggestion of the delineation provided by EXINI for stress polar plots. The third and forth rows show the same delineations for rest polar plots **(g, h, i)** and difference rest-stress plots **(j, k, l)**. The physicians were able to choose between stress, rest or difference polar plots for their delineations.

**Figure 2:**
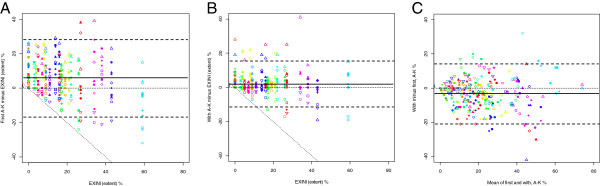
**The relationship between the evaluations made by EXINI (extent) and the physicians, shown as Bland Altman plots.** The left diagram shows the relationship between EXINI and the first evaluation by the physicians **(A)**. “First A-K” on the y axis represents first delineations for the physicians (A-K). The middle diagram shows the relationship between EXINI and the evaluation by the physicians when they were provided with a suggestion by EXINI **(B)**. “With A-K” on the y axis represents the second delineation (with suggestion by EXINI) for the physicians (A-K). The third diagram shows the relationship between the first and second (“with”) delineations made by the physicians **(C)**. The solid line represents mean value, and the heavy dotted lines represent ±1.96 SD. For diagram A and B, the thin dotted diagonal line represents the limit of maximum possible difference (i.e. no value is possible below this line). The twenty-five colours represent the 25 different patients and the 11 symbols represent the 11 physicians (A-K).

For all 25 patients, mean extent obtained from EXINI was 17.0% (SD ± 14.6%). Mean extent for all physicians was 22.6% (± 15.6%) for the first delineation, and 19.1% (± 14.9%) for the second evaluation, where the physicians were provided computerized suggestion of the delineation by EXINI. The difference was statistically significant (p = 0.002). ICC for physicians increased from 0.56 (CI 0.41-0.72) for the first delineation to 0.81 (CI 0.71-0.90) for the second. SD between physicians was 7.8 for the first delineation and 5.9 for the second. The difference between the physicians and EXINI was smaller when the physicians were provided with the suggested delineation by EXINI, except for two physicians, who had a very small deviation from EXINI also for the first delineation. Table 4 shows the differences in mean extent between each physician and EXINI, without and with suggestion by EXINI. Figure 1 shows the delineations made by the 11 physicians and by EXINI for patient #12 without and with suggestions by EXINI. Figure 2 shows the relationship between the evaluation made by EXINI and the physicians (first evaluation and the evaluation with suggestion by EXINI). As seen in the figure, the variability among the physicians varied for small, medium-sized and large ischemic areas. Figure 2A and 2B show that the variability for the reported ischemic areas decreased when the physicians where provided with suggestion by EXINI. Figure 2C shows that for most patients and physicians, the ischemic area was reduced between the first and the second delineation, but for some cases, the area was increased.

**Table 4:** Differences in mean extent values between each physician and EXINI, without and with suggested delineation of the defect made by EXINI

**Physician**	**First evaluation**	**P-value**	**Evaluation with suggestion**	**P-value**
**A**	4.2	0.025	3.3	0.018
**B**	-0.1	0.95	0.8	0.54
**C**	7.4	<0.0001	3.9	0.0049
**D**	7.2	<0.0001	0.4	0.79
**E**	11.3	<0.0001	0.9	0.52
**F**	12.6	<0.0001	3.1	0.027
**G**	-2.2	0.25	0.1	0.95
**H**	-3.5	0.063	-3.5	0.011
**I**	12.6	<0.0001	4.4	0.0016
**J**	19.1	<0.0001	12.5	<0.0001
**K**	-7.0	0.0002	-3.0	0.031

Mean extent value for EXINI was 17.0%.

## Discussion

In this study, we found that the inter-observer variability for the delineation of ischemia was considerable when both regarding different physicians and different software packages. We also found that the inter-observer variability decreased when the physicians were provided with a suggested delineation of the ischemic defects by EXINI.

The results are of value when considering the new recommendation made by the European Society of Cardiology regarding revascularization: patients with stable angina and ischemic amount of myocardium >10% should receive revascularization, whereas patients with ischemic myocardium <10% should receive only medical treatment [6]. Therefore, quantitative analysis of ischemic area is needed to determine the therapeutic strategy for patients with ischemic heart disease. But how can the physician interpreting the MPS images decide on the amount of ischemic myocardium when inter-observer variability among physicians and software packages is large? The recommendation was based on studies by Hachamovitch et al [3,5], where the authors found that in the setting of no or mild amounts of inducible ischemia, patients undergoing medical therapy had a survival advantage over patients undergoing revascularization. These two lines intersected at a value of ≈ 10% to 12.5% myocardium ischemic, above which the survival benefit for revascularization over medical therapy increased as a function of increasing amounts of inducible ischemia. As seen in Figure 2, the variability among the physicians varied for small, medium-sized and large ischemic areas.

It is well known that SSS, summed rest scores (SRS) and SDS differ between different programs. Guner et al [7] compared the diagnostic performances of three different software packages in detecting coronary artery disease by ^201^Tl-MPS. They found that the diagnostic performances of the programs to detect coronary artery disease were similar. However, there were differences in the quantitative values produced by the programs. The differences in SDS between different programs were significant (p < 0.0001). The highest correlation was between 4DM and QPS (r = 0.68), and the lowest was between ECT and QPS (r = 0.41). Wolak et al [8] concluded that there were differences in myocardial perfusion quantification, diagnostic performance, and degree or automation of three software packages with correlations between SSS for the three different software packages evaluated ranging from r = 0.68 to r = 0.84. Svensson et al [9] also investigated the quantification of reversible perfusion defects by three different software packages. They found that widely used software packages commonly differ in their quantification of myocardial perfusion defects and conclude that the interpreting physician should be aware of these differences when using scoring systems.

Not only do the scoring values differ between different software tools, there is also the risk of obtaining scores in areas that are not believed to be ischemic (for example can a patient get many scores of 1 in several areas of the myocardium). The method of delineation of defects by EXINI mimics the physicians’ way to delineate. It is also easier to show the referring physician (often a cardiologist) the extent of the defects rather than scoring values. One should remember, however, that the recommendation of 10% is based on studies using scoring values and not defect extents. Such studies are needed before incorporating the method into clinical routine.

Despite the possible obstacles to provide a percentage of ischemic myocardium to the referring cardiologist, our study has also shown a possible solution. When the physicians were provided with a suggested delineation of the defect, the inter-observer variability decreased. Thus, a program where the physicians easily can adjust the suggested delineation could serve as a tool to make the estimated ischemic amount more standardized. Such a program could, beside standardization, also be used for the training of young doctors.

One advantage for the present study is the relatively large number of physicians involved as well as that the physicians were from several hospitals and countries. Typically, studies involving inter-observer variability include only a few physicians, often from the same department, which tend to decrease inter-observer variability. Thus, the present study shows a “truer” variability in evaluations made by different physicians.

The findings of the present study should be considered in the light of some limitations. In order to find enough physicians willing to participate in the study, a limited number of patients were included. However, it is of greater importance to include several physicians and from different departments/countries. Also, we were not able to verify the evaluations made by the physicians and software packages with an independent reference technique. The physicians did not have any background data regarding the patients, no stress test result, and no information about ejection fraction, left ventricular volumes or transient ischemic dilation. It is possible that the ischemic delineations differ from the ones reported here if such information would have been provided.

## Conclusion

There was a large variability in the estimated ischemic defect size obtained both from different physicians and from different software packages. When the physicians were provided with a suggested delineation obtained by EXINI, the inter-observer variability decreased. Physicians should be aware of the large differences between both different physicians and different software packages when giving the amount of ischemic myocardium to the referring physician.

## Competing interests

LE, KNm and KS are employed by EXINI Diagnostics AB.

## Authors’ contributions

LE and ET were the principal investigators, participated in its design and coordination and drafted the manuscript. PHd participated in the design of the study, performed the statistical analysis and helped drafting the manuscript. SF, PHk, AJ, AK, OL, ML, SM, KNa, EO, SS, LJ and HW collected the data, helped in the design of the study and revised the manuscript. KNm and KS made the custom display of EXINI, participated in the design and coordination and revised the manuscript. All authors read and approved the final manuscript.

## Pre-publication history

The pre-publication history for this paper can be accessed here:

http://www.biomedcentral.com/1471-2342/14/5/prepub
